# Zoonotic bacteria in invasive California Kingsnake *Lampropeltis californiae* from Gran Canaria, Canary Islands, Spain

**DOI:** 10.1371/journal.pone.0334944

**Published:** 2025-10-27

**Authors:** Román Pino-Vera, Néstor Abreu-Acosta, Pilar Foronda

**Affiliations:** 1 Instituto Universitario de Enfermedades Tropicales y Salud Pública de Canarias, Universidad de La Laguna, San Cristóbal de La Laguna, Spain; 2 Departamento de Obstetricia y Ginecología, Pediatría, Medicina Preventiva y Salud Pública, Toxicología, Medicina Legal y Forense y Parasitología, Facultad de Farmacia, Universidad de La Laguna, San Cristóbal de La Laguna, Spain; 3 Programa de Doctorado de Ciencias Médicas y Farmacéuticas, Desarrollo y Calidad de Vida, Universidad de La Laguna, San Cristóbal de La Laguna, Spain; Aga Khan University, PAKISTAN

## Abstract

**Introduction:**

Invasive species can spread pathogens to newly colonised areas and indirectly affect animals and humans. In the Canary Islands territory (Spain), the California kingsnake (*Lampropeltis californiae*) is one of the most relevant invasive species because its predatory habits, however, there is scarce information about the microorganisms they carry and the risk to human health, for that reason, and considering previous data on the pathogens harboured by exotic reptiles in the archipelago, the aim of this study was to analyse the presence of pathogenic bacteria in these animals.

**Methods:**

Fifty *L. californiae* specimens from Gran Canaria Island (Canary Islands, Spain) were examined for zoonotic bacteria. For that purpose, faecal samples were obtained during the necropsy of the animals and inoculated in different selective agar media. If bacterial growth was observed, bacterial colonies were subjected to DNA extraction. The species were confirmed using PCR methods and band pattern comparison.

**Results:**

Almost all, 49 out of 50 (98%), of the studied animals were positive for at least one of the selected bacteria. *Salmonella* spp. (76.0%, 38/50), *Yersinia enterocolitica* (58.0%, 29/50), *Pseudomonas aeruginosa* (42.0%, 21/50), *Campylobacter* spp. (34.0%, 17/50) and *Escherichia coli* virulence genes (*stx* and *eae*) (16.0%, 8/50) were identified.

**Conclusions:**

The presence of well-known zoonotic bacteria in *L. californiae* from Gran Canaria suppose a threat to people that use them as pets, especially children, elderly, and animal handlers, since they cause gastrointestinal symptoms that can lead to severe complications and invasive infections. In addition to that, these colubrids could also spread pathogens to other animals and the environment, adding to the notorious problem of biodiversity losses due to predation of native fauna.

## Introduction

According to the International Union for Conservation of Nature [1], invasive alien species are animals, plants or other organisms that are introduced by humans, either intentionally or accidentally, into places outside of their natural range, negatively impacting native biodiversity, ecosystem services or human economy and well-being. Although not all non-native species harm the environment or other species (including human beings), some of them, defined as invasive, have obvious negative impacts in the colonised areas [2]. Many of the studies around invasive animals are focused on the biodiversity (e.g., predation or resource competition) or economic loss (e.g., crop lost due to pests) but these species can also spread pathogens to new areas and indirectly affect other animals or humans, especially if the infectious microorganisms transmitted and the diseases they cause are not endemic, because the local health system is not prepared for its diagnosis, treatment and/or prevention due to their low prevalence [3–5].

An important entry pathway for the invasive species is the exotic pet trade, defined as the trade of non-domesticated animals for exhibition or companionship purposes, which has become a very lucrative business due to the increasing global demand [6,7]. Of all the exotic pets, reptiles are one of the most popular groups and are becoming more and more common in households all around the world [8,9], these animals can introduce themselves into non-endemic areas after being released by their owners or by escaping and can reduce or eradicate local species populations after their colonisation [10]. In the case of reptiles and amphibians, the pet trade has contributed to the largest number of established non-native species worldwide [6]. Some examples are the red-eared slider (*Trachemys scripta*) or the green iguana (*Iguana iguana*), both of which are often sold as pets [11].

Insular ecosystems are biodiversity hotspots especially vulnerable to invasive species because they harbour highly adapted and unique species with typically small population sizes, low reproductive rates, and a lack of predator defences compared with their continental counterparts [12,13]. The Canary Islands (Spain), located in Norwest Africa, near the coast of Morocco (13^o^23’–18^o^80’ West and 27^o^37’–29^o^24’ North) gather those conditions as well as good weather conditions that promote the settlement of alien species [14]. Up to date, at least 1167 exotic flora and fauna has been notified, of which 289 are considered as invasive or potentially invasive [15] being the California kingsnake (*Lampropeltis californiae*) one of the most relevant because its causing a mass reduction of endemic lizards such as the Gran Canaria giant lizard, *Gallotia stehlini*, the Boettger’s wall gecko, *Tarentola boettgeri*, and the Gran Canaria skink, *Chalcides sexlineatus* [16]. This pet reptile was detected free-living for first time in 1998 in Gran Canaria Island (Canary Islands, Spain) possibly due to household escapes or intentional releases by owners and has established itself in four different nuclei, even in protected natural areas [17].

Despite all the knowledge regarding the threat to endemic fauna of these populations, there is a lack of data on the microorganisms they harbour and their zoonotic potential. Santana-Hernández et al. [18] discovered that California kingsnakes were reservoirs of drug-resistant *Salmonella* spp., which can be dangerous to humans and livestock because their proximity to certain urban areas. However, further investigation must be done to completely understand the role of *L. californiae* in Gran Canaria from public health point of view, especially considering previous works with other exotic wild reptiles in the Canarian archipelago whose results showed a risk of human infection [19,20]. For this reason, the aim of this study was to determine the presence of pathogenic bacteria in the California kingsnake population.

## Materials and methods

**Fig 1:**
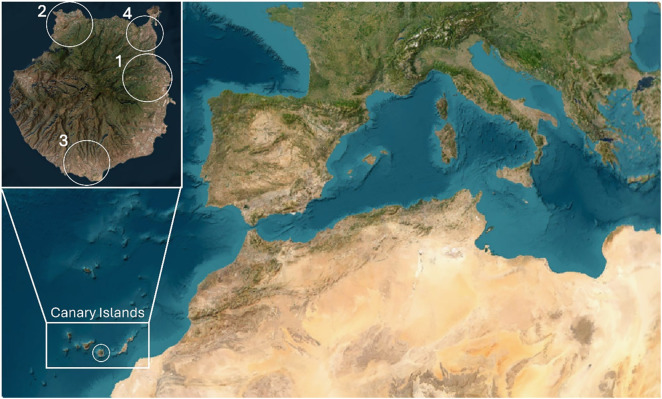
Localisation of Gran Canaria Island (Canary Islands, Spain) and population nuclei of *L. californiae.* 1: primary nucleus, 2: secondary nucleus, 3: tertiary nucleus, 4: quaternary nucleus. Image obtained from USGS Earth Resources Observatory and Science (EROS) Center (Earthshots) [https://eros.usgs.gov/earthshots].

A total of 50 adult California kingsnakes (33 males, 12 females, and 5 sex undetermined) from Gran Canaria (Canary Islands, Spain) (Fig 1) were donated by “Gestión y Planeamiento territorial y ambiental” (Gesplan) and “Red de Alerta Temprana de Canarias para la Detección e Intervención de Especies Exóticas Invasoras” (RedEXOS) staff, who captured and euthanized them under the extermination plan approved by the Canary Islands government. The reptiles were sexed and morphometrical data were taken before the necropsy, during that, faecal matter was obtained for culture, as well as other samples for different studies. The locations of the captured animals are shown in Table 1.

**Table 1:** Localisations of the *Lampropeltis californiae* sampling sites. The nomenclature of the nuclei refers to the date on which they were defined.

Region	nº of snakes
**Primary nucleus**	30
Telde	29
Valsequillo de Gran Canaria	1
**Secondary nucleus**	6
Gáldar	6
**Tertiary nucleus**	4
San Bartolomé de Tirajana	3
Santa Lucía de Tirajana	1
**Quaternary nucleus**	3
Las Palmas de Gran Canaria	3
**Outside defined nucleus**	7
Mogán	1
Las Palmas de Gran Canaria	3
Ingenio	2
Gáldar	1
**Total**	50

### Isolation of bacteria from the samples

A portion of 100 mg of intestinal content from each animal was incubated in 5 mL of Buffered Peptone Water (BPW) (Labkem, Barcelona, Spain) at 37 °C for 24 h except for *Campylobacter* spp. isolation, in this case, the parameters were 42 °C for 18 h applying microaerophilic conditions. After this first incubation, different selective culture media were used: 100 µL of peptone water culture was incubated in Baird–Parker agar (Labkem, Barcelona, Spain) for the isolation of *Staphylococcus* spp., Cetrimide agar (VWR International, Leuven, Belgium) for *Pseudomonas* spp., Cefsulodin Irgasan Novobiocin agar (CIN) (Merck, Darmstadt, Germany) for *Yersinia* spp., Sorbitol supplemented MacConkey agar (Scharlab, Barcelona, Spain) and Tryptone Bile X-glucuronide chromogenic agar (TBX) (Labkem, Barcelona, Spain) for *Escherichia coli*. Every plate was incubated for 24 h at 37 °C except for CIN, which was incubated at 30 °C. In the case of *Salmonella* spp., 500 µL of BPW culture was transferred to 4.5 mL of Rappaport–Vassiliadis Broth (VWR International, Leuven, Belgium) and stored for 20 h at 42 °C. Then, 100 µL of broth culture was later incubated in *Salmonella*–*Shigella* agar (Merck, Darmstadt, Germany) for 24 h at 37 °C.

### Molecular identification

#### DNA extraction.

Five colonies of each culture that matched with the investigated microorganism morphologies were suspended in 1 mL of sterile 1X PBS (pH = 7.4) and centrifuged at 12,000x *g* twice, then the pellet was subjected to DNA extraction following López et al. protocol [21]. For the DNA isolation of *Mycobacterium* spp. and *Campylobacter* spp. 1 mL of BPW culture and 1mL of BPW cultured at 42 °C under microaerophilic conditions respectively were taken and submitted to the same protocol.

#### PCR identification.

Zoonotic bacteria, including resistance and virulence genes, were analysed using different PCR techniques.

A multiplex PCR (m-PCR) with six pairs of primers was employed for *Campylobacter* spp. confirmation and *Campylobacter coli*, *Campylobacter fetus*, *Campylobacter jejuni*, *Campylobacter lari*, and *Campylobacter upsaliensis* identification, according to Wang et al. [22].

Virulence genes *stx*_1_ and *stx*_2_, responsible of Shiga-like toxins synthesis, and *eae*, which codifies for intimin were determined in *E. coli* colonies, following the protocol described by Blanco et al. [23].

Following the process described by Lee et al. [24], a PCR was carried out for the amplification of the *rpoB* gene followed by sequencing for the identification of mycobacterial species.

Cetrimide agar colonies that showed UV fluorescence were tested for *Pseudomonas aeruginosa* through simultaneous amplification of lipoprotein coding genes *oprI* and *oprL*, as described by De Vos et al. [25].

De Freitas et al. [26] protocol was followed for the determination of *Salmonella* serotypes important to human health. It consists of two different PCRs, one to identify *Salmonella* Enteritidis and *Salmonella* Typhi serotypes, and a second one for *Salmonella* Typhimurium serotype detection. In both cases *Salmonella* spp. is also tested.

*Staphylococcus aureus*, *Staphylococcus epidermidis*, *Staphylococcus haemolyticus*, *Staphylococcus hominis*, *Staphylococcus lugdunensis*, *Staphylococcus saprophyticus* and methicillin and mupirocin resistance genes were analysed using a single m-PCR following Campos-Peña et al. [27].

The detection of *ail* (attachment and invasion locus) gene in colonies grown in CIN agar was used for the identification of pathogenic and non-pathogenic *Yersinia enterocolitica* strains, according to Wannet et al. [28].

The results of all PCR assays were evaluated with 1.5% agarose (Fisher Bioreagents, Madrid, Spain) gel electrophoresis at 90V for 1h using SiZer-100 DNA Marker (iNtRON Biotechnology, Seongnam-Si, Republic of Korea) as molecular size marker and Real-Safe (Durviz SL, Valencia, Spain) as DNA stain. The gels were revealed with a ChemiDocTM XRS+ (Bio-Rad, Hercules, CA, USA) system.

### Co-infection index (Ic)

The co-infection index (Ic) developed by Ginsberg [29] is used to quantify the deviation of the number of mixed infections from independence, in other words, it indicates if one pathogen facilitates or hinders the infection of another pathogen. This index is expressed as the difference between the number of co-infections and the expected number due to chance alone, as a percentage of the totality of the infected animals. It follows the next formula:


$$Ic\;=\;\frac{O\;-\;E}{N}\times \text{1}\text{0}\text{0}$$

Where O = number of observed co-infections; E = expected number of *L. californiae* with coinfections due to chance alone; N = total number of *L. californiae* infected by either or both microorganisms. E is calculated with the following formula:


$$E\;=\frac{\left(a\;+\;b)(a\;+\;c\right)}{\left(a\;+\;b\;+\;c\;+\;d\right)}\;;\;N\;=\;a\;+\;b\;+\;c$$

Where a = number of kingsnakes infected by both bacteria (equals O); b = number of kingsnakes infected only with microorganism 1; c = number of kingsnakes infected only with microorganism 2; and d = number of kingsnakes not infected with microorganism 1 nor 2. A positive Ic value means that the number of real co-infections is greater than the expected, and less if it is negative. The significance of the co-infection index was calculated by chi-square test.

### Statistical analysis

Chi-square test and Fisher’s exact test were applied with a stabled *p*-value of 0.05 were applied to compare the prevalence between sexes of the studied animals using the statistical Windows software “SPSS” 29.0.1.0 (IBM Corporation, Armonk, NY, USA). Due most of the animals were captured within the primary nucleus, localization was not considered for statistics. The 95% Clopper Pearson confidence intervals (95% CI) were evaluated using the approximate or exact method, as appropriate.

### Ethics statement

The specimens used in this study were part of a program for eradicating invasive species managed by the Government of the Canary Islands (Spanish Royal Degree 216/2019 and Order 336/20). All the procedures carried out in this work were performed in accordance with Directive 2010/63/EU EEC for animal experiments.

## Results

Forty-nine out of fifty (98.0%, 95% CI: 89.4–99.9) of the studied animals were positive for at least one of the selected bacteria. The most frequently identified pathogens were *Salmonella* spp., found in thirty-eight animals (76.0%, 95% CI: 61.8–86.9), followed by *Y. enterocolitica* and *Pseudomonas* spp. in twenty-nine (58.0%; 95% CI: 43.2–71.8) and twenty-two (44.0%, 95% CI: 30.0–58.7) snakes, respectively. On the contrary, *Mycobacterium* spp. and *Staphylococcus* spp. were not found in any sample. Table 2 shows the identity of all positive samples.

**Table 2:** Isolated bacteria in *Lampropeltis californiae* from Gran Canaria (Canary Islands, Spain) regarding its localization.

Bacteria	Localization	+/*n* (Prevalence %, CI 95%)
***Campylobacter* spp.**	Primary nucleus Secondary nucleus Tertiary nucleus Quaternary nucleus Outside defined nucleus Total	11/30 (36.7%, 19.9–56.1) 1/6 (16.7%, 0.4–64.1) 2/4 (50.0%, 6.8–93.2) 1/3 (33.3%, 0.8–90.6) 2/7 (28.6%, 3.7–71.0) 17/50 (34.0%, 21.2–48.8)
***E. coli* (*stx***_**1**_**, *stx***_**2**_ **and *eae* genes)**	Primary nucleus Secondary nucleus Tertiary nucleus Quaternary nucleus Outside defined nucleus Total	6/30 (20.0%, 7.7–38.6) 2/6 (33.3%, 4.3–77.7) 0 0 0 8/50 (16.0%, 7.2–29.1)
***Mycobacterium* spp.**	Primary nucleus Secondary nucleus Tertiary nucleus Quaternary nucleus Outside defined nucleus Total	0 0 0 0 0 0
***Pseudomonas* spp.**	Primary nucleus Secondary nucleus Tertiary nucleus Quaternary nucleus Outside defined nucleus Total	12/30 (40.0%, 22.7–59.4) 2/6 (33.3%, 4.3–77.7) 1/4 (25.0%, 0.6–80.6) 1/3 (33.3%, 0.8–90.6) 6/7 (85.7%, 42.1–99.6) 22/50 (44.0%, 30.0–58.7)
***Salmonella* spp.**	Primary nucleus Secondary nucleus Tertiary nucleus Quaternary nucleus Outside defined nucleus Total	22/30 (73.3%, 54.1–87.7) 6/6 (100%, 54.1 - 100) 2/4 (50.0%, 6.8–93.2) 1/3 (33.3%, 0.8–90.6) 7/7 (100%, 59.0 - 100) 38/50 (76.0%, 61.8–86.9)
***Staphylococcus* spp.**	Primary nucleus Secondary nucleus Tertiary nucleus Quaternary nucleus Outside defined nucleus Total	0 0 0 0 0 0
***Y. enterocolitica***	Primary nucleus Secondary nucleus Tertiary nucleus Quaternary nucleus Outside defined nucleus Total	19/30 (63.0%, 43.9–80.1) 3/6 (50.0%, 11.8–88.2) 3/4 (75.0%, 19.4–99.4) 2/3 (66.7%, 9.4–99.2) 2/7 (28.6%, 3.7–71.0) 29/50 (58.0%, 43.2 - 71.8)

+: number of positive samples, *n*: total of animals.

### *Campylobacter* spp.

Of the five *Campylobacter* species investigated in this study only three were found: *C. upsaliensis* in one male snake (2.0%; 95% CI: 0.05–10.6), and *C. lari* and *C. coli* in another two (4.0%; 95% CI: 0.5–13.7, one male and one female), remarkably those two animals were co-infected with both *Campylobacter* species. Fourteen isolates (28.0%; 95% CI: 16.2–42.5), four females and ten males, with no significant differences between them, were positive for this bacterium genus but could not be identified at species level with the specific primers used in this study.

### *Escherichia coli* virulence genes (*stx*_1_, *stx*_2_ and *eae*)

*Escherichia coli* carrying *stx* genes were found in eight animals (16.0%; 95% CI: 7.1–29.1). Four (8.0%; 95% CI: 2.2–19.2) positive isolates for *stx*_2_, two (4.0%; 95% CI: 0.5–13.7) for *stx*_1_ and two (4.0%; 95% CI: 0.5–13.7) with both genes coexisting in the same specimens, however *eae* gene were not identified in any sample. The results according to sex are described in Table 3, no statistical differences were obtained.

**Table 3:** Number and percentage of *Escherichia coli* virulence genes (stx_1_, stx_2_ and eae) found in *Lampropeltis californiae* from Gran Canaria (Canary Islands, Spain).

Virulence genes	Positive males (prevalence, CI 95%) *n* = 33	Positive females (prevalence, CI 95%) *n* = 12	Undetermined *s*ex positives *n* = 5
***stx***_**1**_	2 (6.1%, 0.7–20.2)	0	0
***stx***_**2**_	4 (12.1%, 3.4–28.2)	0	0
***stx***_**1**_ **and *stx***_**2**_	1 (3.0%, 0.1–15.8)	1 (8.3%, 0.2–38.5)	0
***eae***	0	0	0
**Total**	7 (21.2%, 9.0–38.9)	1 (8.3%, 0.2–38.5)	0

### *Pseudomonas* spp.

**Fig 2:**
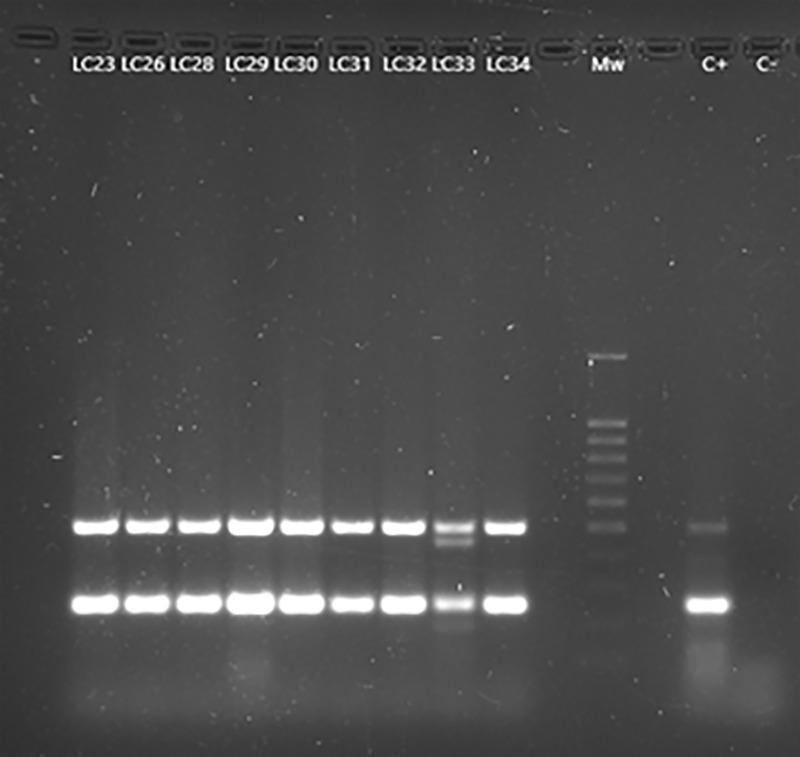
PCR results for the detection of *Pseudomonas aeruginosa.* LC 23-34 lanes: oprI (249 bp) and oprL (504 bp) genes amplification fragments, characteristic of *P. aeruginosa*. Mw: molecular size marker (SiZer-100 DNA Marker, iNtRON Biotechnology). C + : positive control. C-: negative control. Non marked lanes do not contain any sample.

Twenty-one (42.0%; 95% CI: 28.1 –56.8) snakes were positive for *P. aeruginosa* (Fig 2), fourteen males and seven females, without significative differences. Additionally, another one (2.0%; 95% CI: 0.05–10.6) isolate was positive for a different *Pseudomonas* species.

### *Salmonella* spp.

*Salmonella* was the most frequent bacterial genus found in this study, with thirty-eight out of fifty (76.0%, 95% CI: 61.8–86.9) kingsnakes being positive (Table 4). Of those thirty-eight isolates, fourteen (28.0%, 95% CI: 16.2–42.5) correspond to *S*. Typhimurium, seven (14.0%, 95% CI: 5.8–26.7) to *S*. Typhi and three (6.0%, 95% CI: 1.3–16.5) to *S*. Enteritidis serotypes, whereas nineteen (38.0%, 95% CI: 24.7–52.8) positive samples could only be identified as *Salmonella* sp. Co-infection with different serotypes were observed: *S*. Enteritidis and *S*. Typhimurium were detected in one (2.0%, 95% CI: 0.05–10.6) specimen as well as *S*. Typhi and *S*. Typhimurium in four (8.0%, 95% CI: 2.2–19.2) ones. There were not significative differences between males and females.

**Table 4:** Number and percentage of *Salmonella* spp. and *Salmonella enterica* serotypes found in *Lampropeltis californiae* from Gran Canaria (Canary Islands, Spain).

Serotype	Positive males (prevalence, CI 95%) *n* = 33	Positive females (prevalence, CI 95%) *n* = 12	Undetermined *s*ex positives (prevalence, CI 95%) *n* = 5
***S*. Enteritidis**	2 (6.1%, 0.7–20.2)	0	1 (20.0%, 0.5–71.6)
***S*. Typhi**	5 (15.2%, 5.1–31.9)	2 (16.7%, 2.1–48.4)	0
***S*. Typhimurium**	8 (24.2%, 11.1–42.3)	5 (41.7%, 15.2–72.3)	1 (20.0%, 0.5–71.6)
***Salmonella* spp.** ^**†**^	16 (48.5%, 30.8–66.5)	1 (8.3%, 0.2–38.5)	2 (40.0%, 5.3–85.3)
**Total** ^‡^	28 (84.8%, 68.1–94.9)	7 (58.3%, 27.7–84.8)	3 (60.0%, 14.7–94.7)

†Serotypes different from *S.* Enteritidis, *S.* Typhi and *S.* Typhimurium, ‡Considering the co-infections.

### Yersinia enterocolitica

**Fig 3:**
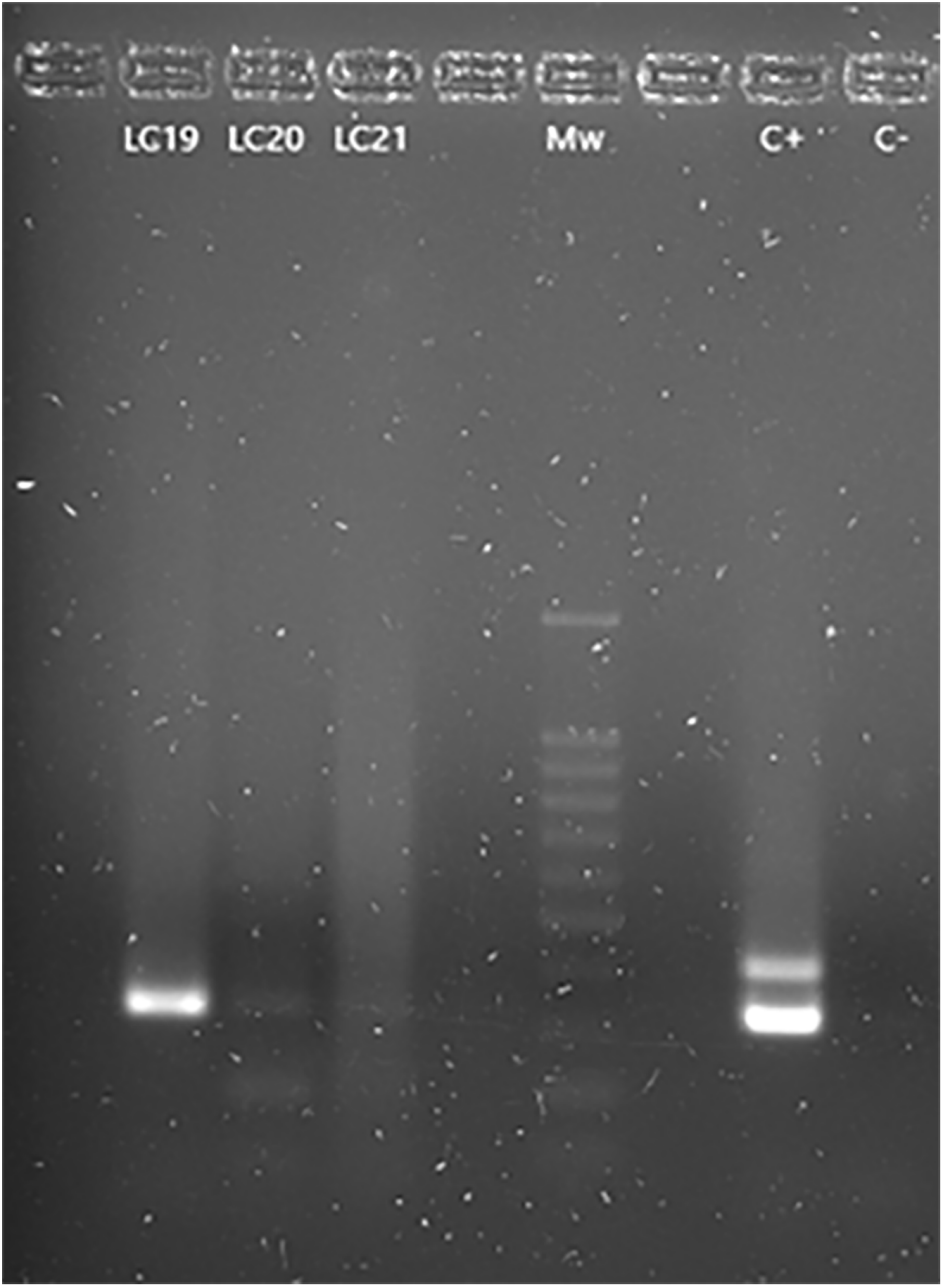
PCR results for the detection of *Yersinia enterocolitica.* LC 19 and 20 lanes: 16S rRNA gene (330 bp) amplification fragments corresponding to *Y. enterocolitica*. LC21: negative sample Mw: molecular size marker (SiZer-100 DNA Marker, iNtRON Biotechnology). C + : positive control (the 425 bp (ail gene) fragment belongs only to pathogenic strains) C-: negative control. Non marked lanes do not contain any sample.

*Yersinia enterocolitica* was found in twenty-nine (58.0%, 95% CI: 43.2–71.8) animals, twenty (60.6%, 95% CI: 42.1–77.1) males, 6 (50.0%, 95% CI: 21.1–78.9) females and 3 (60.0%, 95% CI: 14.7–94.7) undetermined sex, showing no significative differences between them. Nevertheless, the *ail* gene, which is commonly found in pathogenic strains, was only present in three (6.0%, 95% CI: 1.3–16.5) of them, with two (6.1%, 95% CI: 0.7–20.2) males and one (20.0%, 95% CI: 0.05–71.6) being a snake of undetermined sex (Fig 3).

### *Mycobacterium* spp. and *Staphylococcus* spp.

None of the snakes sampled were positive for *Mycobacterium* spp. or *Staphylococcus* spp.

### Co-infections and Co-infection Index (Ic)

From all the positive animals identified in this study, nine of them (18.0%, 95% CI: 8.6–31.4) were infected by just one investigated pathogen, nineteen (38.0%, 95% CI: 24.7–52.8) hosted two pathogens, twelve (24.0%, 95% CI: 13.1–38.2) hosted three different bacteria, seven (14.0%, 95% CI: 58.2–26.7) animals were infected with four pathogens and two (4.0%, 95% CI: 0.5–13.7) snakes hosted six pathogens. The most frequent co-infections are described in the table below (Table 5). All co-infections can be consulted in S1 Table.

**Table 5:** Most frequent co-infections found in *Lampropeltis californiae* from Gran Canaria (Canary Islands, Spain).

Identified bacteria/virulence genes	Number of animals with co-Infections	Percentage (%) of co-Infections (*n* = 213)^†^
***Salmonella* spp. + *Y. enterocolitica***	23	10.8
***Salmonella* spp. + *P. aeruginosa***	15	7.0
***Campylobacter* spp. + *Salmonella* spp.**	12	5.6
***Campylobacter* spp. + *Y. enterocolitica***	12	5.6
***Campylobacter* spp. + *P. aeruginosa***	9	4.2
***P. aeruginosa* + *Y. enterocolitica***	9	4.2
***S.* Typhimurium + *Y. enterocolitica***	9	4.2
***P. aeruginosa *+* S.* Typhimurium**	7	3.3
***E. coli* carrying *stx***_**2**_ **gene + *Y. enterocolitica***	5	2.3
***E. coli* carrying *stx***_**2**_ **gene + *Salmonella* spp.**	5	2.3
***Campylobacter* spp. + *S.* Typhimurium**	5	2.3
***Salmonella* spp. +* E. coli* carrying *stx***_**1**_ **gene**	4	1.9
**Pathogenic *Y. enterocolitica *+ *Salmonella* spp.**	3	1.4
***E. coli* carrying *stx***_**1**_ **gene + *Y. enterocolitica***	3	1.4
**S. Enteritidis + *Y. enterocolitica***	3	1.4

†Number of co-infections with two different bacteria.

Co-infection indexes and statistical test results for que most frequent co-infections are shown in Table 6. The *p* – values obtained, all above 0.05, indicates that none of the pathogens involved increase or decrease the chance of co-infection between themselves.

**Table 6:** Co-infection indexes and p-values of the most frequent co-infections found in *L. californiae* from Gran Canaria (Canary Islands, Spain).

Identified bacteria	Co-infection index (Ic)	*p* – value
***Salmonella* spp. + *Y. enterocolitica***	2.2	0.520
***Salmonella* spp. + *P. aeruginosa***	−2.2	0.520
***Campylobacter* spp. + *Salmonella* spp.**	−2.1	0.728
***Campylobacter* spp. + *Y. enterocolitica***	6.3	0.238
***Campylobacter* spp. + *P. aeruginosa***	6.4	0.261
***P. aeruginosa* + *Y. enterocolitica***	−7.8	0.065
***S.* Typhimurium + *Y. enterocolitica***	2.6	0.752
***P. aeruginosa *+* S.* Typhimurium**	4.0	0.475
***E. coli* carrying *stx***_**2**_ **gene + *Y. enterocolitica***	5.1	0.380
***E. coli* carrying *stx***_**2**_ **gene + *Salmonella* spp.**	1.1	1.000
***Campylobacter* spp. + *S.* Typhimurium**	0.9	1.000
***Salmonella* spp. +* E. coli* carrying *stx***_**1**_ **gene**	2.5	0.560
**Pathogenic *Y. enterocolitica *+ *Salmonella* spp.**	1.9	1.000
***E. coli* carrying *stx***_**1**_ **gene + *Y. enterocolitica***	2.3	0.630
***S.* Enteritidis + *Y. enterocolitica***	4.3	0.254

## Discussion

### *Campylobacter* spp.

Campylobacteriosis is one of the most common causes of gastroenteritis and the most reported zoonosis in the world, with an increasing tendency over the last years in all countries, *C. jejuni* being the main bacterium isolated, followed by *C. coli* in second place, even though, other less studied species had been found affecting humans [30,31]. This disease courses with unspecific symptoms such a diarrhea, fever or stomach cramps but can develop complications such as irritative bowel and Guillain-Barré syndromes [32]. Studies have reported that the increasing popularity of pet squamates is globally attached to a rising incidence of campylobacteriosis over the last decade together with evidence that squamates may harbour *Campylobacter* spp. and are able to transfer them to humans through contaminated food and water, pet handling or cross-contamination through their faeces [33].

In this study three animals were positive, two for both *C*. *coli* and *C*. *lari* along with one snake with *C. upsaliensis*, however, *C. jejuni* was not identified. These three bacteria are a genetically close group of thermotolerant bacteria with an optimal growth temperature of 42 °C whose truly clinical and public health relevance, except for *C. coli*, stills indetermined because of the lack of clinical laboratories that identify *Campylobacter* spp. infections to species level [34]. The low prevalence of these species in *L. californiae* could be due their high grow temperature and the ectothermic characteristics of the host snakes that also have shadow-living habits. Apart from those positives results, more *Campylobacter* spp. isolates were identified only at genus level because the primers used for PCR identification were not specific for less frequent or non-zoonotic species. The predominating *Campylobacter* species in reptiles are *Campylobacter fetus* subsp. *testudinum*, *Campylobacter iguaniorum* and *Campylobacter geochelonis* [35]. Therefore, probably these unidentified bacteria belong to the reptile-related *Campylobacter* spp., considering that, a posterior study with different primers should be done to determine the species. Also, it may be noticed that the positive isolates for *C. upsaliensis*, *C. lari* and *C. coli* could coexist with different species not tested in the study because solid agar medium was not used.

Comparing with previous studies in other countries, the 34.0% (*n* = 50) *Campylobacter* spp. prevalence obtained in this study is similar to the prevalence obtained in snakes from zoos and private owners of The Netherlands (32.0%, 32/100) [36]. Nonetheless these two results differ significantly with another one from Northern Italy, with all the 45 snakes tested were negative [37]. In Taiwan, one study analysing *C. fetus* in reptiles found 5.0% (*n* = 20) of positive samples in Pythonidae, Colubridae and Boidae faecal samples [38].

*Campylobacter* spp. presence has been also studied in other reptiles such as chelonians, turtles and tortoises, with low prevalence, in eastern Spain, 517 samples from 200 native and exotic turtles did not show any positive results [39] and in Nothern Italy only 5 (1.1%) out of 452 samples were positive [40]. The reason of this difference could be the higher detection rate of PCR method in comparison of cultivation [41].

### *Escherichia coli* virulence genes (*stx*_1_, *stx*_2_ and *eae*)

*Escherichia coli* is broadly distributed in the environment and can be commonly found as normal microflora in the intestine of mammals and birds. However, some strains can be pathogenic depending on the presence of different virulence factors and cause severe diseases such epidemic dysentery, neonatal meningitis or haemolytic uraemic syndrome [42,43]. Shiga-like toxin is related to *E. coli* pathogenicity as it is considered the most relevant virulent factor. This toxin is encoded in *stx* genes, which are divided in two types: *stx*_1_ and *stx*_2_, with the second one more involved in severe symptoms. In another way, intimin protein encoded by *eae* gene is important for the pathogenicity of virulent *E. coli* due to its role in the attaching of the bacterium to the intestinal epithelium [44,45].

Because of being commonly associated and more prevalent in warm-blooded animals including humans, *E. coli* has been poorly described in reptiles [46–48]. Few studies were found about the presence of this bacterium and even less analysing virulence factor genes. In Poland, 60.4% (58/96) and 35.5% (12/34) of juvenile and adult European pond turtles (*Emys orbicularis*) respectively were positive for *E. coli* [49] and another work in Turkey obtained 25 out of 150 (16.7%) positive *E. coli* isolates from snake-eyed lizards (*Ophisops elegans*) cloacal swab samples [50].

The scarce data available shows that *E. coli* is significatively prevalent in reptiles isolates but the presence of *stx*_1_, *stx*_2_ and *eae* genes is diverse. Martínez et al. [51] did not detect Shiga-like *E. coli* in 20 ocellated lizards (*Timon Lepidus*) from a research centre in southwest. Moreover, none of the 67 faecal samples from different reptiles analysed by Dec et al. [52] in Poland presented *stx* or *eae* genes, despite that 32 (47.8%) were positive for this bacilli, with no significant difference between snakes, lizards and turtles, while half of them (16) contained other virulence factor genes, for example *traT* and *fyuA* that codifies the complement resistance protein and the ferric yersiniabactin uptake receptor respectively. However, Bautista-Trujillo et al. [53] collected 240 faecal samples from *I. iguana* which 62 (25.9%) were carrying diarrheagenic *E. coli* with *stx*_1_ gene appearing more frequently than *stx*_2_ and *eae* genes (in order, 10.0%, 0.42% and 0.8%). In comparison, *L. californiae* from Gran Canaria have similar *stx*_1_ (8.0%) and *eae* (0.0%) prevalences but *stx*_2_ gene (12.0%), related with serious disease, was significantly higher.

This first approach gives basic information about the *E. coli* pathogenicity in California kingsnakes, but more studies involving other virulence factors apart from *stx* and *eae* genes are needed to determine the importance of these animals in *E. coli* infections.

### *Mycobacterium* spp.

Mycobacterial infection in reptiles is less frequent than in warm-blooded animals, within this animal group, chelonians are more commonly infected while it is very rare in snakes, with only a few reports since the last century [54,55]. Different species can cause mycobacteriosis in reptiles, mostly belonging to atypical (non-tuberculous) mycobacteria complex like *Mycobacterium avium*, *Mycobacterium chelonae* or *Mycobacterium fortuitum*, in any case, the transmission pathways in these animals is not completely known. It appears that mycobacteria enter the reptile body through ingestion or skin injuries and cause granulomatous lesions in different organs that can lead to systemic infection in some cases [56,57]. In this study, none of the 50 *L. californiae* sampled were positive for *Mycobacterium* spp. and did not show any macroscopic lesion during the necropsies, which is consistent with the low number of reports of these bacteria in snakes. In contrast Ebani et al. [58] found that 13 out of 18 (72.2%) captive snakes were positive for *Mycobacterium* spp. showing that this bacterium could cause mostly asymptomatic infections in snakes and that *L. californiae* from Gran Canaria appears not to be in contact with this pathogen.

### *Pseudomonas* spp.

Bacteria belonging to *Pseudomonas* genus can be found worldwide in different environments, including soil, water, air, plants, animals and humans due to their metabolic and physiologic versatility, with relevance in plant and human illness and potential in biotechnological applications [59,60]. From all the species, *P. aeruginosa* probably is the most studied one because of its importance in human health since it is an opportunistic pathogen responsible of many nosocomial infections and the bacterium most frequently found colonising medical devices such as catheters or ventilators using its capacity to form biofilms. It is responsible of skin, blood, urinary and pulmonary infections and have special relevance in chronic respiratory diseases like chronic obstructive pulmonary disease (COPD) or cystic fibrosis [61,62].

Different animals can act as reservoirs of *Pseudomonas* due to its high adaptability; studies have detected its presence in wild boars, mallards, ticks, farm animals, pets, rabbits, micromammals, deers and freshwater fish and identified different species apart than *P. aeruginosa* such as *Pseudomonas putida* or *Pseudomonas fluorescens*, [63,64]both unfrequently causes of skin and soft tissue infections (SSTIs) and bacteremia, mostly in immunodeficient patients [65].

In reptiles, *Pseudomonas* spp. has also been found. Ebani et al. [66] examined 218 faecal samples of healthy pet reptiles from Italian households which 22 (10.1%) were infected with *Pseudomonas* spp. Another study comparing the presence of *P. aeruginosa* in various snake species found a significative difference between captive animals from France and wild ones from Guinea with 72 out of 83 (87.7%) positive samples in the first case and 3 out of 23 (13.0%) in the second one [67], the authors stands for the geographical distribution of the wild reptiles, apart from human activities, as the reason of the low prevalence. In Northern Italy 251 out 419 (59.9%) cloacal swab samples from farm snakes were positive for *P. aeruginosa*, with statistical differences between taxonomic families, being Boidae snakes the most prevalent (35/45; 77.8%), following by Pythonidae (196/326; 60.1%) and Colubridae (20/48; 41.7%) [68]. This last result is very similar to the obtained in *L. californiae* of Gran Canaria (42.0%) and could indicate an increased immune response against this bacterium in comparison with other snakes’ families, or because the preferred temperature of colubrids do not allow *P. aeruginosa* to multiplicate properly.

### *Salmonella* spp.

*Salmonella* is one of the major foodborne bacterial pathogens worldwide. In humans it causes gastroenteritis (salmonellosis) with typical symptoms such as diarrhoea, fever, and abdominal cramps but occasionally can lead to an invasive infection [69]. Most of human cases are associated with the ingestion of contaminated water or food from animal sources like eggs, milk and meat, although direct contact transmission has been described, both person-to-person and animal-to-person [70,71]. The nomenclature of the *Salmonella* genus is complex and have undergone many changes from its first description. Today, this taxon is divided into two species: *Salmonella bongori* and *Salmonella enterica*, and seven subspecies: I, II, IIIa, IIIb, IV, V, and VI. Apart from that there are over 2500 serotypes with different host adaptability and pathogenicity with most serotypes belonging to *Salmonella enterica* subsp. *enterica* (subspecies I) and responsible of 99% of salmonellosis in warm-blooded animals, including humans [72,73].

*Salmonella* spp. is commonly found asymptomatically in the intestinal tract of reptiles with approximately 40% of the serotypes being host-specific and rarely found in other animals. Although these serotypes can infect humans, most of the reptile-associated salmonellosis are caused by less specific serotypes [74–76]. Reptile-associated human infections are frequently caused by *Salmonella* Typhimurium, *Salmonella* Enteritidis and *Salmonella* Paratyphi, this last one capable of causing enteric fever [77,78].

In this study the presence of *Salmonella* spp. in *L. californiae* was relatively high (76.0%, 38/50), with 24/50 (48.0%) positive isolates for well-known human pathogenic serotypes: *S*. Typhi (*n* = 7), *S*. Enteritidis (*n* = 14) and *S*. Typhimurium (*n* = 3), highlighting the risk of transmission of these snakes, taking account their proximity to populated areas. The identification of *S.* Typhi serotype is interesting considering not only its relationship with typhoid fever, that can be deadly, but its establishment as a human-host restricted bacterium [79]. This could mean that *L. californiae* in Gran Canaria lives in faecal contaminated areas and could be acting as a carrier of this dangerous bacterium. Remarkably, a recent study done by Santana-Hernández et al. [18] in the same host and island obtained a smaller prevalence of *Salmonella* spp. (20.5%, 15/73) and no positive samples for *S*. Typhi, *S*. Enteritidis nor *S*. Typhimurium. The reasons of this difference could be the geographic area, time of sampling (as shedding is intermittent), or methodology used, as the authors mentioned in their article.

Regarding other studies outside the Canary Islands, in Poland, 10 out of 45 (22.2%) free-living grass snakes (*Natrix natrix*) were positive for *Salmonella* spp. but no further information about subspecies or serotypes was given [80]. In Croatia, 20 wild four-lined snakes (*Elaphe quatuorlineata*) studied were negative for *Salmonella* spp. [81] and, in Germany, 23 European adders (*Vipera berus*), 12 grass snakes and 21 slow worms (*Anguis fragilis*) were analysed looking for various bacteria with *Salmonella* spp. being only found in 8 (34.8%) of the adders, concretely *S. enterica* subsp. *diarizonae* (IIIb) [82]. Similar to the occurred with *P. aeruginosa* prevalence, wild snakes show lower positivity rates than domestic snakes (from pet shops, zoos or private households), in mainland Spain, some studies have been done with different kind of reptiles in captivity: Marin et al. [83] detected *S. enterica* in 59 out of 123 (48.0%) of the animals sampled, with significant differences between snakes (76.0%, 16/21) and lizards (69.0%, 33/48), compared to chelonians (19.0%, 10/54). This last result is almost equal to the obtained in other work of the same author where 29 out of 152 (19.1%) tortoises and turtles studied were positive for this bacterium, with the mention that all the 71 turtles (pond and sea turtles) were negative, highlighting the terrestrial habits of this bacterial genus [84]. This higher prevalence in snakes comparing to other reptilian families taxa have been also noticed in other studies such as Hydeskov et al. [85], which found that snakes from Copenhagen zoo were infected by *S. enterica* (63.3%, 33/53), followed by chelonians (36.4%, 24/66) and lizards (14.8%,12/81) or Bjelland et al. [86], with 62% (*n* = 53) and 67% (*n* = 15) of *Salmonella* spp. positive snakes and lizards respectively, against 3% (*n* = 15) of infected chelonians from three zoos in Norway. In both two studies, all the differences were statistical significance except between the Norwegian snakes and lizards.

The high prevalence of *Salmonella* spp. found in this study is similar to other ones referred to domestic snakes but, considering that *L. californiae* was captured in the wilds it could be sign of human presence near the population area or it treats of a new population with many individuals from households’ escapes.

### *Staphylococcus* spp.

Some *Staphylococcus* species are part of the normal cutaneous and mucosal microbiota of humans and animals but can act as opportunistic pathogens, especially in individuals with deficient immune system [87]. *Staphylococcus aureus* is the most frequent cause of human infection because of its virulence and antibiotic resistance, followed by less common species such as *S. epidermidis*, *S. hominis* or *S. saprophyticus*, these three species belonging to the coagulase-negative staphylococci species group [88]. In reptiles, bacteria belonging to *Staphylococcus* genus are frequently found in their skin, but its clinical relevance depends on many factors such the skin integrity, the host species or the coexistence with different microorganisms [89]. Other studies have isolated *Staphylococcus* spp. from the oral cavity of some reptiles causing illness like ulcerative stomatitis and involving a risk of secondary infection to humans due to bites or incorrect handling [90,91] but only a few have analysed faeces or cloacal swab samples with mostly low prevalences, for example, Espinosa-Gongora et al. [92] did not isolate any positive sample for *S. aureus* in any of the 21 reptiles studied (including lizards, tortoises and snakes) from the Copenhagen zoo, while another study, focused on exotic pet animals from the Iberian Peninsula, found only 11 out of 345 (3.2%) reptiles carrying *Staphylococcus* spp. and just one positive for *S. aureus* (0.3%) [93]. These data match with the negative results found in this study, suggesting that *L. californiae* from Gran Canaria are not involved in the transmission of human pathogenic *Staphylococcus* spp., however, the bacterial growth in Baird Parker agar indicated that the snakes could be carrying *Staphylococcus* species less common in human infections that were not part of the PCR protocol used like, for example, *Staphylococcus pseudointermedius* [94].

### Yersinia enterocolitica

*Yersinia enterocolitica* is the third cause of bacterial diarrhoea in Europe and the main etiologic agent of enteric yersiniosis followed, but much less frequent, by *Yersinia pseudotuberculosis*. Most of the infection occurs in children under 10 years of age with clinical manifestations such as diarrhoea, fever, and abdominal pain, however, systemic forms can be found in elderly persons and patients with certain underlying conditions affecting the skin, throat, lungs, liver and/or kidney even leading to sepsis [95,96].

This species compounds a heterogenous group of strains that can be found in the intestinal tract of a huge variety of animals or the environment, being the pigs the main reservoirs of human pathogenic strains while most of the environmental samples has been described as non-virulent [97,98]. Enteropathogenic *Y. enterocolitica* carries, among others, the *ail* gene which encodes a small outer membrane protein that facilitates the attachment and invasion of host cells and provide serum resistance [99]. Most of the studies found about this bacterium are referred to warm-blooded animals, while data regarding reptile infection is very scarce. Only a few articles were found, showing very low prevalence or even absence of *Y. enterocolitica* in reptile-origin samples, however, most of these studies were made decades ago [100–103] and their results could not be reliable nowadays because of the improvement of detection and identification methods or the increased animal trade and human-animal contact. Nevertheless, more recent works [49,104] have also described low prevalences of *Y. enterocolitica* in reptiles, in contrast with the 58.0% (29/50) obtained in this study, indicating a close (direct or indirect) contact between the *L. californiae* population sampled and warm-blooded animals or remarkable susceptibility to *Y. enterocolitica* infection. In any case, only three of the twenty-nine positive isolates presented the *ail* gene, meaning that the risk of human transmission is low, even so, more studies searching for *Y. enterocolitica* or other human pathogenic *Yersinia* species in reptiles must be done to understand the real threat of reptile-origin yersiniosis.

### General discussion

Almost all *L. californiae* individuals (49/50, 98.0%) investigated were positive for at least one pathogenic bacterium capable of causing human infection. The most frequent species was *S. enterica* (serotypes *S*. Typhi, *S*. Typhimurium and *S*. Enteritidis), followed by *Y. enterocolitica* and *P. aeruginosa*. These high infection rates in these wild snake populations, which are normally lower than in domestic ones, could indicate a close contact with humans, probably due to faecal contamination of the habitats. Besides that, the household origin of *L. californiae* in Gran Canaria could be another reason of the high prevalences, if the pathogen can maintain its life cycle within the population even in wild conditions. Also, the diet of the California kingsnake, that consists mainly of lizards and rodents [105] could have relation with the results obtained, for example *G. stehlini*, an endemic lizard that habits the island, is known as a great carrier of *Salmonella* spp. [106], however, more studies must be done regarding this kind of transmission.

Multiple coinfections were observed, from all the 49 infected animals 40 were hosting two or more pathogens at the same time, however, the statistical analysis made showed no correlation between the bacteria species meaning that none identified microorganism facilitates or difficult the infection of another one and all coinfections are due to chance alone. Further investigation must be done to evaluate the risk and severity of human transmission of these combinations. Also no statistical significative differences of the infection rates between sexes were observed.

This study is among the first ones that look for zoonotic bacteria in *L. californiae* from Gran Canaria, for this reason, more studies need to be done with a wider range of pathogens of both different species not previously investigated and other serotypes or species of the confirmed isolates, i.e., *Salmonella* serotypes or coagulase-negative staphylococci as well as antibiotic resistance trials and a greater number of specimens. Even if this snake species is not considered aggressive to humans [107] it could be interesting to analyse oral swabs since many studies have confirmed the presence of zoonotic bacteria in the oral cavity of different snakes and confirmed the possibility of secondary infection after bites [108,109]. The results obtained in this study highlight the risk posed to human health *by L. californiae* populations in Gran Canaria, especially to people with deficient immune system and animal handlers because of their close contact with the snakes and their environment.

## Conclusions

Several bacteria with relevance to human and animal health were found in California kingsnake (*L. californiae*) populations studied in the island of Gran Canaria (Canary Islands, Spain), among all the positive isolates, zoonotic *S. enterica* serotypes, enterotoxigenic *E. coli* and pathogenic *Y. enterocolitica* suppose the greatest threat to people, especially children, elderly, and animal handlers. In addition to that, these colubrids could also spread pathogens to other animals and the environment, adding to the well-known problem of biodiversity loss due to predation of native fauna.

Considering the results obtained and their importance to public health, more studies need to be done with larger sample sizes and different microbiological essays to fully understand the epidemiology of the of the pathogenic bacteria present in the invasive *L. californiae* in the Canary Islands.

## Supporting information

S1 Table Number of California kingsnakes (*Lampropeltis californiae*) from Gran Canaria (Canary Islands, Spain) with co-infections found in this study. (XLSX)

S1 File Raw gels. (PDF)
