# Supplementary material for: Zoonotic bacteria in invasive California Kingsnake Lampropeltis californiae from Gran Canaria, Canary Islands, Spain
Source: PLoS One. 2025 Oct 27;20(10):e0334944. doi: 10.1371/journal.pone.0334944 (PMC12558559; doi:10.1371/journal.pone.0334944)
Supplement: S1 File — (PDF) [file pone.0334944.s002.pdf]

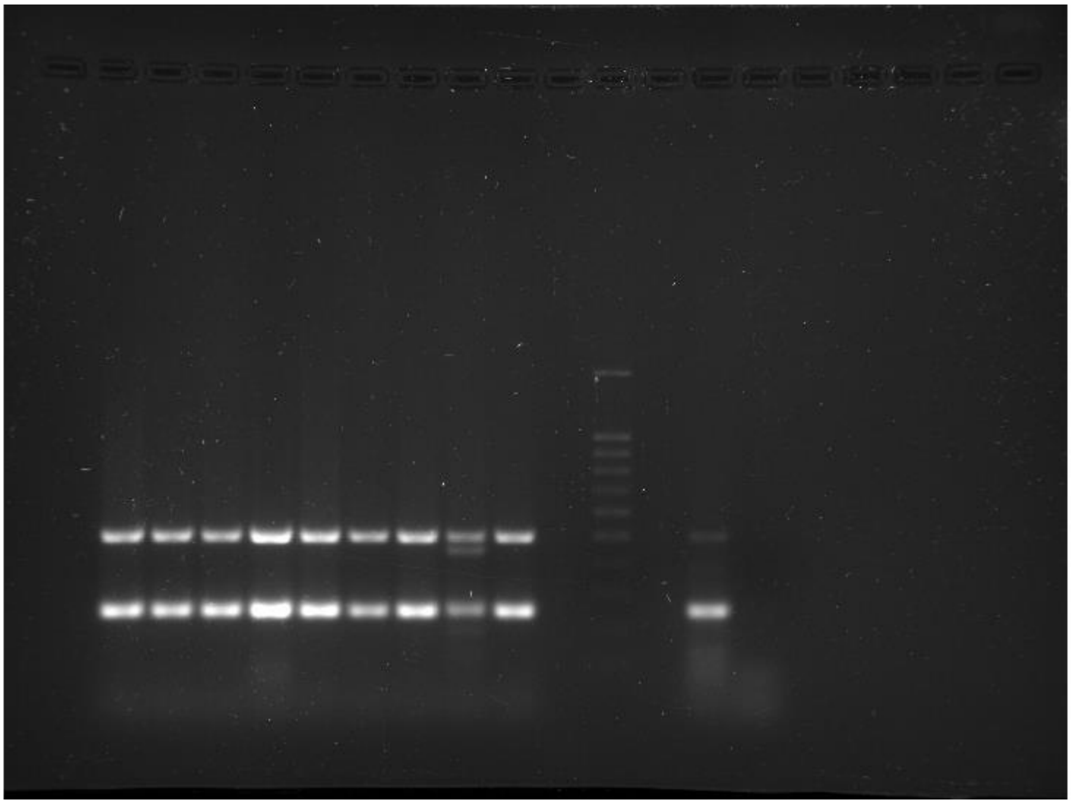

**Fig 2. PCR results for the detection of *Pseudomonas aeruginosa*.** LC 2-10 lanes: *oprI* (249 bp) and *oprL* (504 bp) genes amplification fragments, characteristic of *P. aeruginosa*. Lane 12: molecular size marker (SiZer-100 DNA Marker, iNtRON Biotechnology). Lane 14: positive control. Lane 15: negative control. The rest of the lanes do not contain any sample. The image was captured using a ChemiDocTM XRS+ (Bio-Rad, Hercules, CA, USA) software.

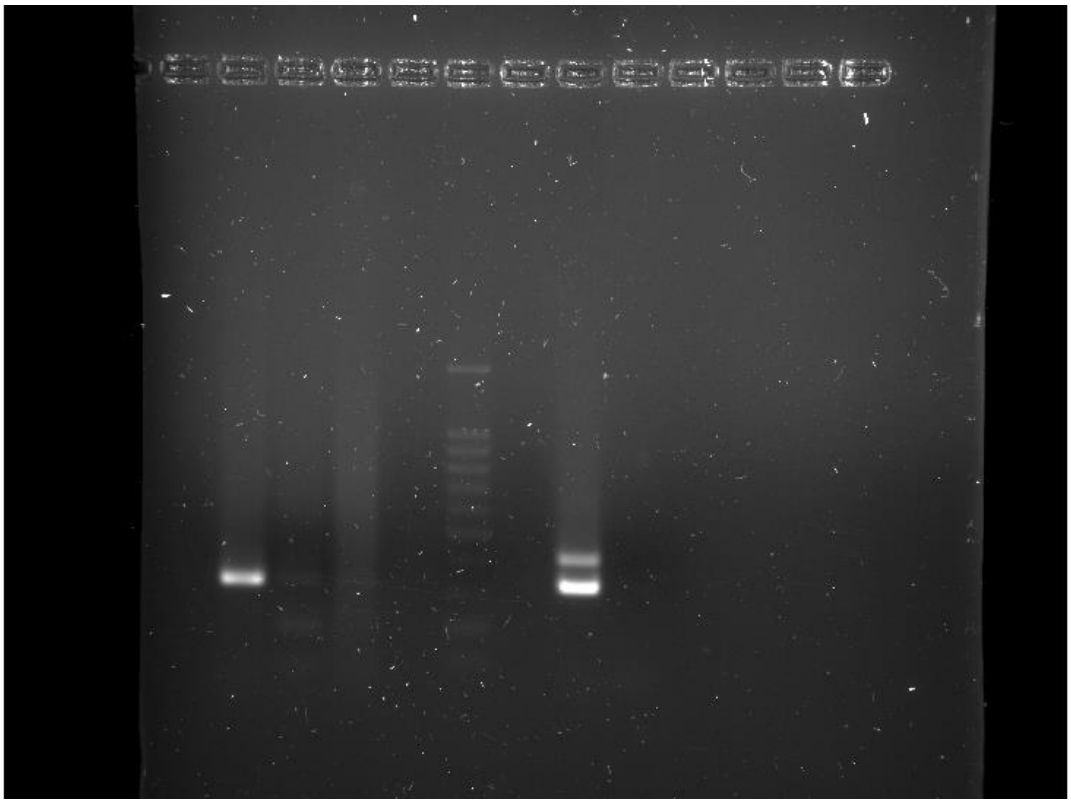

**Fig 3. PCR results for the detection of *Yersinia enterocolitica*.** lanes 2 and 3: 16S rRNA gene (330 bp) amplification fragments corresponding to *Y. enterocolitica*. Lane 4: negative sample. Lane 6: molecular size marker (SiZer-100 DNA Marker, iNtRON Biotechnology). Lane 8: positive control (the 425 bp (*ail* gene) fragment belongs only to pathogenic strains) Lane 9: negative control. The rest of the lanes do not contain any sample. The image was captured using a ChemiDocTM XRS+ (Bio-Rad, Hercules, CA, USA) software.
